# Decreased Opioid Consumption in Bone Marrow Harvest Patients Using Quadratus Lumborum Blocks in a Standardized Protocol

**DOI:** 10.3389/fmed.2022.862309

**Published:** 2022-04-26

**Authors:** Nicole C. McCoy, Ellen L. Hay, Deborah A. Romeo, J. Wesley Doty, Bethany J. Wolf, Michelle P. Hudspeth

**Affiliations:** ^1^Anesthesia and Perioperative Medicine, Medical University of South Carolina, Charleston, SC, United States; ^2^Department of Public Health Sciences, Medical University of South Carolina, Charleston, SC, United States; ^3^Pediatric Hematology/Oncology, Medical University of South Carolina, Charleston, SC, United States

**Keywords:** regional anesthesia, opioid analgesics, nerve block, postoperative pain, local anesthesia, quadratus lumborum, bone marrow harvest

## Abstract

**Purpose:**

Bone marrow harvesting is associated with significant postoperative pain that may have potential negative consequences for the patient and health care system. In the current absence of uniform guidelines, there exists considerable variability amongst providers with respect to perioperative analgesia, especially opioid administration. In this initiative, we explored the potential for preoperative bilateral quadratus lumborum blocks in combination with a standardized perioperative analgesic protocol to manage pain with the goal of reducing perioperative narcotic usage and thereby improving opioid stewardship.

**Methods:**

Adults who underwent bone marrow donation from 2018 to 2020 were included in this analysis (*n* = 32). The pre-implementation group (*n* = 19) was reviewed retrospectively while the implementation group (*n* = 13) was evaluated prospectively. Patient demographics, pain scores, and opioid consumption were evaluated.

**Results:**

Patient characteristics were equivalent except for anesthesia type with an increased number of patients in the implementation group undergoing spinal anesthesia. The implementation group showed significantly reduced median intraoperative (20.0 mg vs. 0.0 mg; *p* < 0.001) and total opioid consumption (20.5 mg vs. 0.0 mg; *p* < 0.001). The number of patients who received any opioids decreased from 84.2% (16/19) before implementation to 23.1% (3/13) after implementation.

**Conclusion:**

This change in practice suggests that implementation of a standardized perioperative protocol, including bilateral quadratus lumborum blocks, for bone marrow harvest patients leads to reduced perioperative opioid administration without compromising immediate perioperative pain control.

## Introduction

Bone marrow harvesting (BMH) is a safe and effective method for collecting hematopoietic stem cells (1, 2). Clinicians often prefer bone marrow over mobilized peripheral blood stem cells because bone marrow has a lower risk of graft vs. host disease (2–4). Bone marrow donation is a critical step in treating certain blood disorders and cancers, such as leukemia and aplastic anemia, often resolving the disease process for recipients.

Because pain is often the most common postoperative complication (5, 6), potential donors are concerned about post-procedural pain (5, 6). To manage pain during the procedure, donors are placed under general or spinal anesthesia, and then given opioids intraoperatively as needed, infiltrated local anesthesia at the surgical site at the end of the procedure, and intravenous analgesics including opioids in the post anesthesia care unit (PACU) (7–10). Donors are also given oral opioids to take at home because, during the first 12–48 h after BMH, they can experience significant pain at their surgical site (11–14). Without adequate control, this pain can lead to inpatient admission (14–16), delayed return to work, and reduced quality of life (17, 18). Also, if donors need higher doses of opioids to manage pain, they may experience unwanted side effects (19), such as impaired cognition and an inability to perform at preoperative levels (20). Worse yet, opioid overuse may predispose patients to opioid dependence or misuse (21, 22). These risks may prevent people from donating bone marrow (5, 6).

At our institution, we noticed differing amounts of opioids were administered to patients intraoperatively, whether prophylactically or triggered by a change in vital signs, during BMH. We also noticed that patients were treated with intravenous or oral opioids in the PACU, despite infiltration of local anesthetic at the end of BMH. Based on these observations, we sought alternative approaches to managing pain after BMH, improving opioid stewardship, and reducing provider variability in treating pain.

In this quality improvement (QI) initiative, we set out to create a standardized protocol through multiple tests of change that would reduce variability between providers and thereby produce consistent perioperative outcomes. We also explored alternative approaches to treating postoperative pain, including quadratus lumborum (QL) fascial plane blocks. These procedures are low risk and have been used to successfully treat postoperative pain in a variety of procedures, including lower abdominal surgery, hip arthroplasty, caesarian section, pelvic fractures, and lumbar laminectomies (23). We postulated that QL blocks could replace infiltration of local anesthetic and, when paired with a standardized protocol, would reduce perioperative opioid administration in adult patients undergoing BMH.

## Methods

### Patient Population

All patients were adults 18 years and older who underwent BMH at our academic medical institution. This study was designed as a QI analysis of our practice and approved by the Institutional Review Board at the Medical University of South Carolina. From 2018 to 2020, a total of 46 patients underwent BMH at our institution; patients under the age of 18 were excluded from the analysis. The baseline group (*n* = 19) included adults undergoing BMH between January 31, 2018, and January 21, 2020. Data collection for the baseline group was collected retrospectively. The implementation group (*n* = 13) included patients undergoing BMH between January 22, 2020, and October 6, 2020. Data for the implementation group was collected prospectively. Table 1 summarizes the demographic data and clinical characteristics of the two groups.

**TABLE 1:** Patient and procedural characteristics.

Patient and procedural characteristics	Before implementation (*n* = 19)	After implementation (*n* = 13)	*P*-value
Age, years, median (min, max)	30 (21, 61)	29 (22, 45)	0.519
**Sex, *n* (%)**			0.513
Male	11 (57.9)	9 (69.2)	
Female	8 (42.1)	4 (30.8)	
Weight, kg, median (min, max)	83.0 (61.0, 152.7)	89.5 (58.1, 149.1)	0.985
**Donor type, *n* (%)**			0.243
Parent	2 (10.5)	3 (23.1)	
Child	7 (36.8)	1 (7.69)	
Sibling	5 (26.3)	3 (23.1)	
NMDP	5 (26.3)	6 (46.2)	
**ASA, *n* (%)**			0.467
I	12 (63.2)	10 (76.9)	
II	7 (36.8)	3 (23.1)	
**Anesthesia type, *n* (%)**			0.002
GETA	19 (100.0)	7 (53.9)	
Spinal	0 (0.0)	6 (46.1)	
Total duration, minutes, median (min, max)	154 (91, 265)	160 (73, 272)	0.71
Procedure duration, minutes, median (min, max)	102 (38, 213)	94 (24, 205)	0.732
GETA, minutes, median (min, max)	102 (38, 213)	104 (93,205)	0.494
**Spinal, minutes, median (min, max)**		66 (24, 111)	
PACU duration, minutes, median (min, max)	52 (45, 245)	125 (43, 363)	0.015
GETA, minutes, median (min, max)	52 (45, 245)	51 (43, 128)	0.308
**Spinal, minutes, median (min, max)**		184 (93, 363)	
Harvest volume, mL, median (min, max)	1,350 (235, 2,300)	1,600 (410, 2,630)	0.359
**Pain score reported, *n* (%)**			0.141
Yes	11 (57.8)	11 (84.6)	
No	8 (42.2)	2 (15.4)	
If Yes, pain score, median (min, max)	4 (1, 7)	0 (0, 10)	0.068
Harvesting physicians, # unique	5	2	NA
Anesthesiologist, # unique	15	5	NA

*ASA, American Society of Anesthesiologists; GETA, general endotracheal anesthesia; NA, not applicable; NMDP, National Marrow Donor Program.*

### Quality Improvement Framework

**FIGURE 1:**
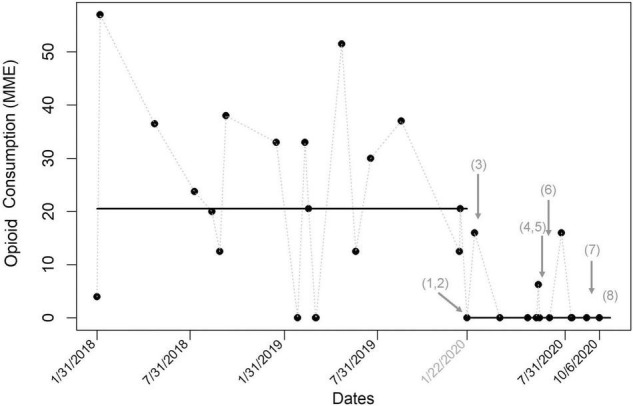
Run chart for total oral morphine milligram equivalents (MME) in bone marrow donors (*N* = 32). The line represents median MME. Multiple tests of change over time are noted with arrows. After implementing the protocol and quadratus lumborum (QL) block, administered MMEs significantly decrease. This decrease was maintained over time and through multiple tests of change, supporting the sustainability of the protocol. The tests of change included: (1) instituted QL blocks, (2) created anesthesia quality improvement champion, (3) standardized preoperative multimodal medications, (4) formalized perioperative protocol, (5) added anesthesia attendings to staff cases, (6) standardized intraoperative multimodal medications, (7) reduced postoperative oxycodone prescriptions, and (8) uploaded formal protocol to the anesthesia electronic medical record. BMT, bone marrow transplant.

To reduce perioperative opioids and improve postoperative pain control, we developed a standardized protocol that was continually assessed and improved over approximately 9 months. During this time, we implemented QL fascial plane blocks to manage pain. We also developed strategies to deliver multimodal preoperative and intraoperative medications, and to eliminate infiltration of local anesthetic by BMH physicians. To improve opioid stewardship, we implemented processes to enhance communication with the regional anesthesia pain service (RAPS) to limit or avoid using opioids during the QL blocks and when inducing anesthesia (if safe/possible), and to eliminate standardized opioid orders in the PACU. To decrease provider variability, we designated an anesthesia physician liaison to manage donors perioperatively, limited the anesthesia attendings who staffed the cases, and created a standardized protocol for anesthesia and BMH physicians to reference. The timeline for implementing these tests of change is outlined in Figure 1. A key drivers diagram is presented in Supplementary Figure 1 detailing the various components of the quality improvement aim. Supplementary Figure 2 outlines the final protocol that incorporated each perioperative phase and recommendations for anesthetic management.

At the beginning of the project, we identified several stakeholders, including the QI physician champion, harvesting physicians, RAPS physicians, and perioperative nursing leadership. The QI physician champion became the anesthesia physician liaison to the bone marrow transplant team. This team included two harvesting physicians who consistently performed all bone marrow harvests starting September 16, 2019 (a decision made by the BMH team before implementing this QI initiative in January 2020 but continued with adherence throughout). The lead QI physician champion discussed incorporating regional anesthesia options with the bone marrow transplant team at their weekly meeting before starting a new protocol or changing to an established practice. The anesthesia liaison and nurse coordinator for the bone marrow transplant team also communicated and collaborated during the project.

### Quadratus Lumborum Block

All patients undergoing BMH in the implementation period underwent lateral QL blocks bilaterally before being transported to the operating room. A lateral QL technique was chosen because the approach is the most superficial and easiest to visualize with ultrasound. For the block, the patient was most often in the supine position. Using ultrasound, the QL muscle was identified and 20 mL of ropivacaine (0.375–0.5%) was deposited between the trans vs. abdominis aponeurosis and the QL muscle. This process was repeated on the opposite side. The RAPS attending provided sedation as needed, which consisted of midazolam, dexmedetomidine, and, infrequently, fentanyl.

### Data Collection

To assess pain control, we used perioperative opioid use as a proxy measure. Perioperative opioid use included intraoperative intravenous opioids given by the anesthesia team based on vital sign chances consistent with pain and intravenous or oral opioids given in the PACU based on patients’ description of pain. The primary calculation excluded intravenous opioids given to sedate patients for the preoperative QL block or as part of inducing anesthesia to blunt a hemodynamic response to direct laryngoscopy. Opioid administration in all phases of care was collected for both groups and converted to oral morphine milligram equivalents (MME) using a standardized equianalgesic dosage conversion calculator.^1^ Patient demographics, anesthesia type, procedure duration, PACU duration, and PACU pain scores were analyzed in each group.

### Statistical Analysis

The median, inner quartile range, and range for intraoperative MME, PACU MME, and total MME received by donors before and after implementation were calculated. The median was selected as the distribution of MME’s was skewed. Univariate associations were evaluated with categorical variables using the Fisher’s exact test, and associations with continuous variables were evaluated using the Wilcoxon rank sum test. All analyses were conducted in SAS v. 9.4 (SAS Institute, Cary, NC) (24).

## Results

### Patient Characteristics

Patient demographics and clinical characteristics for the groups before and after implementation are reported in Table 1. Patient age, sex, weight, donor type, American Society of Anesthesiologists status, procedure duration, total duration of harvest, and harvest volume did not significantly differ before and after implementation. Reported pain scores did not significantly differ between groups although 42% of pain scores were not reported in PACU documentation in the baseline group. After implementation, significantly more participants received spinal anesthesia than general endotracheal anesthesia (*p* = 0.002) which was driven by protocol change. PACU times were also significantly longer in the implementation group (52 min vs. 125 min; *p* < 0.015) attributable to the patients recovering from the spinal anesthetic.

### Opioid Use

**FIGURE 2:**
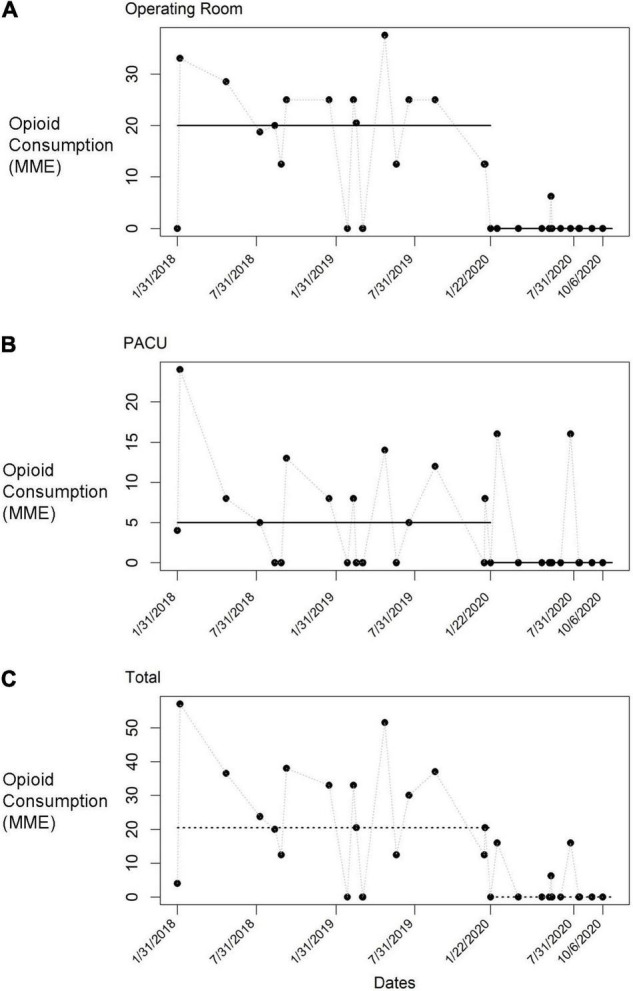
Run charts of opioid consumption before implementation and throughout multiple tests of change. Each data point represents an individual donor. **(A)** Morphine milligram equivalents (MME) administered in the operating room. **(B)** MME administered in the post anesthesia care unit (PACU). **(C)** MME administered in all phases of care.

The median intraoperative, PACU and total opioid consumption by time are shown in Table 2. After implementation, the median intraoperative and total opioid consumption significantly decreased (*p* < 0.001 for both parameters). PACU consumption also decreased after implementation, though the change was not statistically significant (*p* = 0.073). The number of patients who received any opioids decreased from 84.2% (16/19) before implementation to 23.1% (3/13) after implementation. In all cases, median opioid consumption dropped to zero after implementation. Figure 2 shows run charts of total, intraoperative, and PACU opioid consumption over time.

**TABLE 2:** Summary of opioid consumption in oral morphine milligram equivalents.

	Prior to implementation, median (IQR; min-max) (*n* = 19)	After implementation, median (IQR: min-max) (*n* = 13)	*P*-value
Intraoperative MME	20.0 (12.5; 0–37.5)	0.0 (0.0; 0–6.25)	<0.001
PACU MME	5.0 (8.0; 0–24)	0.0 (0.0; 0–16)	0.073
Total MME	20.5 (22.3; 0–57)	0.0 (0.0; 0–16)	<0.001

*IQR, interquartile range; MME, morphine milligram equivalents; PACU, post anesthesia care unit.*

Among all patients in the implementation group, only one received an intravenous opioid mid-procedure to treat delirium. The patient underwent spinal anesthesia and infusion of propofol. He was treated with one dose of fentanyl to keep him still and comfortable during the procedure. As his spinal level of anesthesia was adequate for the procedure, opioids were not administered to manage procedural pain.

In the PACU, only two patients received intravenous opioids. These patients underwent longer harvests with larger total harvest volumes, which can increase postoperative pain (9). Interestingly, both patients underwent general anesthesia with an endotracheal tube and supplemental ketamine infusions. Shortly into their PACU stay, both patients reported non-specific pain and were treated with opioids. In follow-up calls, both patients reported low pain scores in the first 48 h after the procedure and that their opioid use was minimal due to their perceived pain control from the nerve blocks.

## Discussion

In this QI initiative, we introduced a novel use of a well-established procedure, QL blocks, to successfully manage postoperative pain. We also standardized the process for perioperative anesthetic management, educated care team members on the appropriate use of opioids, initially decreased variability between anesthesia providers, and increased new provider involvement with excellent adherence to an accessible provider protocol. During this approximately 9-month initiative, we significantly and sustainably reduced opioids given to patients undergoing BMH, implicating that our initiative improved pain control in these patients.

Early in project development and based on ongoing revisions of the perioperative protocol due real-time outcomes, we surprisingly found that the quadratus lumborum blocks and multimodal medications provided sufficient analgesia during the procedure. After this was repeatedly observed, we made a continued effort to educate providers on the benefit of the components of the protocol and the potential lack of need for additional opioid administration in the operating room. This was particularly true for patients undergoing general anesthesia, who did not have anticipated vital sign changes secondary to pain with incision despite lack of intravenously administered opioids. This observed principle was not applicable for patients undergoing spinal anesthesia, as incisional pain was masked by the spinal level of analgesia therefore intravenous opioids were unlikely to be given to these patients.

Our results support the growing body of literature that describes QL blocks as an effective modality for pain management of the lower back. The distribution of the blockade among all anatomic variations of QL blocks is cited as T7-L2 (23, 25). The lateral QL block has been shown to consistently anesthetize T12-L1 (26–28). Importantly, for procedures of the posterior iliac crest, the dermatome involved is T11- T12, and the osteotome involved is L1-L2. Additionally, QL blocks have an excellent safety profile. Cited complications of QL blocks are due to known complications of fascial plane blocks. For example, local anesthetic could spread to the lumbar plexus, causing temporary weakness of the lower extremities. Also, local anesthetic systemic toxicity could occur, as with all regional anesthesia techniques, so appropriate rescue medications should be available when a regional block is performed. Finally, the proximity of the pleura and kidney may pose a risk for needle trauma, but this risk is rare when using ultrasound guidance to perform the blocks (23).

In QI work, the success of new processes may depend on one person who can consistently oversee the process, or on effective protocols that minimize variability between personnel (29). One success of our project was that the care team adhered to the written protocols, even without direct oversight. This success illustrates that with effective communication between the anesthesia liaison and care team, and the use of a standardized protocol, the process can become hardwired and sustainable.

We recognize that we cannot fully eliminate perioperative opioid administration. Opioids are often needed for complex cases (30). The complexity depends on a variety of factors, including preexisting patient-related challenges, block failure, duration of harvest, volume of harvest, and lack of administration of additional adjuncts (i.e., multimodal medication adjuncts). Although we would not deny patients opioids, our initiative shows that QL blocks and a standardized protocol in combination are a sustainable approach to significantly reducing opioid use in the perioperative timeframe. The significant decrease in intraoperative opioids was derived from the observed success of the QL blocks as an intraoperative pain management modality and the recommendations from the protocol for conservative opioid administration. We had previously observed liberal use of opioids in our practice, given prophylactically and to treat vital sign changes indicative of pain. This may be seen as a limitation as the reduction was due to the analgesic effect of the block plus the elective oversight to reduce opioid use but if patients were exhibiting post procedural pain, there should be an increase in opioid administration in the PACU. Additionally, in our primary data analysis we did not include opioids that were given during the induction of general anesthesia as the pre-implementation group had a 100% administration rate. The implementation group was comprised of patients who underwent general anesthesia and spinal anesthesia therefore the groups did not undergo similar induction methods. The rate of administration of opioids for induction of general anesthesia in the implementation group (*n* = 7) was 0%. Therefore, inclusion of the induction opioid would have further increased the observed difference between the two groups. Supplementary Figure 1 illustrates the oral MME per patient when induction opioids were included and excluded.

A notable barrier encountered was the lack of buy-in from nurses in the PACU who managed patient recovery after spinal anesthesia. Because donors who underwent spinal anesthesia had a longer recovery time in the PACU (median duration 184 min), their care required more time from nurses, especially with staffing constraints during the pandemic. Although shorter-acting spinal medications could have addressed this issue, these medications were not readily available due to production delays. This issue also highlights the importance of including a broad spectrum of stakeholders in multi-departmental QI projects. Another barrier that others could face is the lack of a regional anesthesia–specific service to perform the QL blocks. To address this issue, the supervising anesthesiologist could perform the blocks in the operating room either before inducing anesthesia or between inducing anesthesia and starting the surgery.

Our study is not without potential limitations. The project was designed as a QI analysis of our practice comparing a prospective patient group with a retrospective data set. Additionally, a power calculation was not performed. In QI initiatives the primary aim is not to show a statistical difference in the groups but to improve the quality of a process or patient experience. Finally, decreased opioid administration alone may not be an indication of decreased pain.

In future work, we plan to develop a perioperative protocol specific to our pediatric donors and to obtain formal postoperative satisfaction surveys. Also, we are now performing BMH on adult-related donors in operating rooms at the children’s hospital. This effort aims to improve patient satisfaction by limiting their transport across our medical campus after their procedure and before their family member’s bone marrow transplant within the same day. We will also continue to evaluate and improve our ambulatory spinal protocol. Although general and spinal anesthesia have similar safety margins, each approach may have unique benefits. Specifically, in this patient population, spinal anesthesia causes less postoperative nausea and vomiting, whereas general anesthesia provides airway protection (8). Midway through the project, we stopped routinely using a spinal anesthetic for the procedure because PACU staff were concerned about prolonged PACU stays. In the future, we would prefer to use a different spinal medication to shorten the duration of the block on appropriate patients, thereby reducing their stay in the PACU.

## Conclusion

In this initiative, we used a standardized protocol and QL blocks to reduce variability between providers and significantly reduce perioperative opioid use related to BMH. Our approach also improved perioperative pain control in patients undergoing the procedure. These findings may alleviate donor concerns about pain associated with BMH. This project highlights another successful application of QL blocks for pain management, further supports their use in procedures involving posterior iliac crests and reinforces the role of regional anesthesia in opioid sparing anesthetics.

## Data Availability Statement

The original contributions presented in the study are included in the article/Supplementary Material, further inquiries can be directed to the corresponding author/s.

## Ethics Statement

The studies involving human participants were reviewed and approved by QI Analysis via the IRB at the Medical University of South Carolina. Written informed consent for participation was not required for this study in accordance with the national legislation and the institutional requirements.

## Author Contributions

NM designed the project, collected and evaluated data, and was the primary author for the manuscript. EH was involved in the development of the project, data collection, and manuscript revisions. DR helped with data evaluation and manuscript revisions. JD helped the manuscript and subsequent revisions. BW helped with the statistics and results. MH helped with project design, mentored our team, and was involved in manuscript revisions. All authors contributed to the article and approved the submitted version.

## Conflict of Interest

The authors declare that the research was conducted in the absence of any commercial or financial relationships that could be construed as a potential conflict of interest.

## Publisher’s Note

All claims expressed in this article are solely those of the authors and do not necessarily represent those of their affiliated organizations, or those of the publisher, the editors and the reviewers. Any product that may be evaluated in this article, or claim that may be made by its manufacturer, is not guaranteed or endorsed by the publisher.

## Footnotes

1
https://clincalc.com/Opioids/

## Supplementary Material

Supplementary Figure 1 Key drivers diagram illustrating change ideas, key drivers and overall project aim.

Supplementary Figure 2 Standardized protocol for patients undergoing bone marrow harvest.

Supplementary Figure 3 Run chart for total oral morphine milligram equivalents (MME) in bone marrow donors (*N* = 32). The line represents the median MME. Induction opioids are included for all patients as noted by the red data points. Induction opioids are excluded as noted by the black data points.
