# Supplementary figures and images for: Decreased Opioid Consumption in Bone Marrow Harvest Patients Using Quadratus Lumborum Blocks in a Standardized Protocol
Source: Front Med (Lausanne). 2022 Apr 26;9:862309. doi: 10.3389/fmed.2022.862309 (PMC9086676; doi:10.3389/fmed.2022.862309)

Supplemental Figure 1.

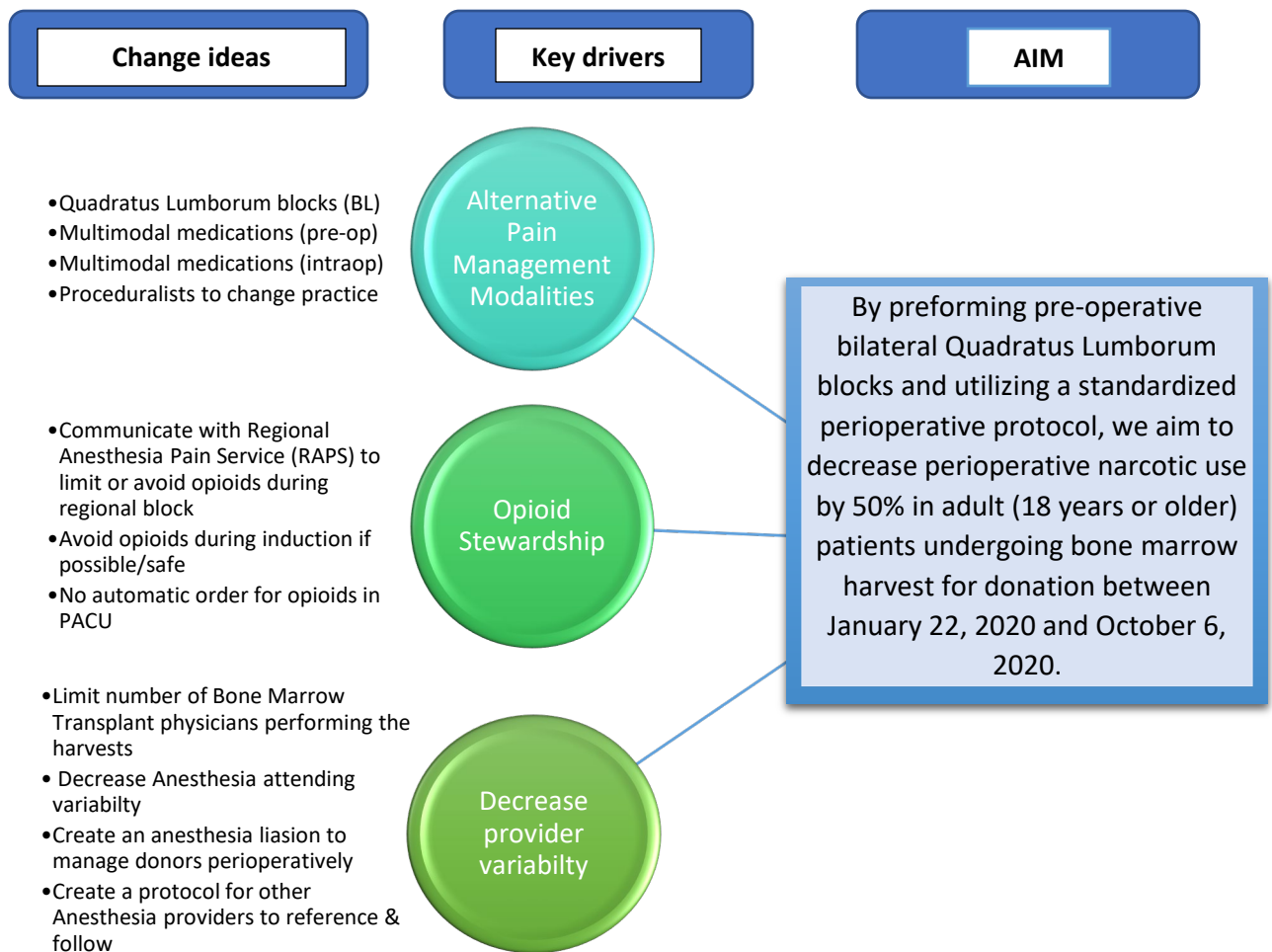

Supplement: Supplementary Figure 1 — Key drivers diagram illustrating change ideas, key drivers and overall project aim. [file Image_1.PDF]

Supplemental Figure 3.

A. Operating Room

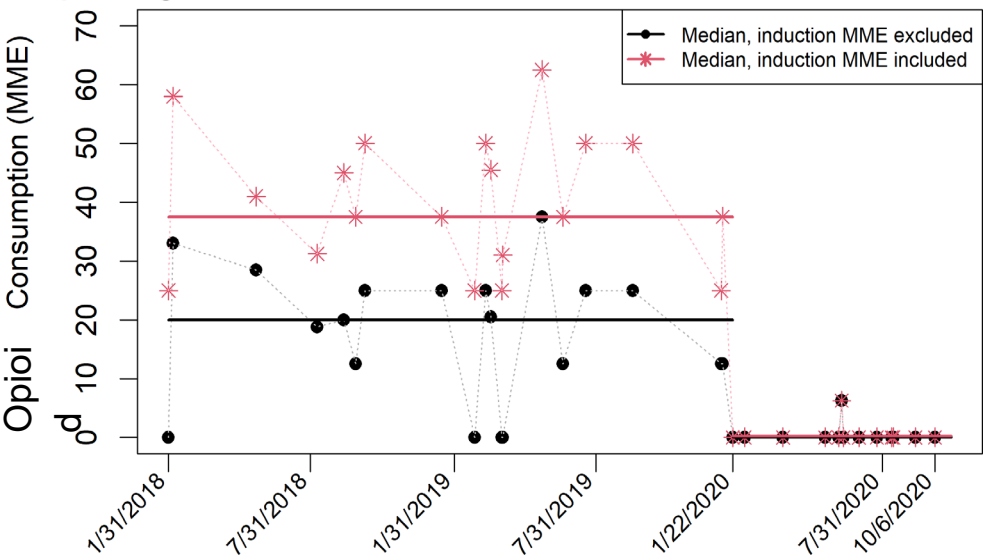

B. Total

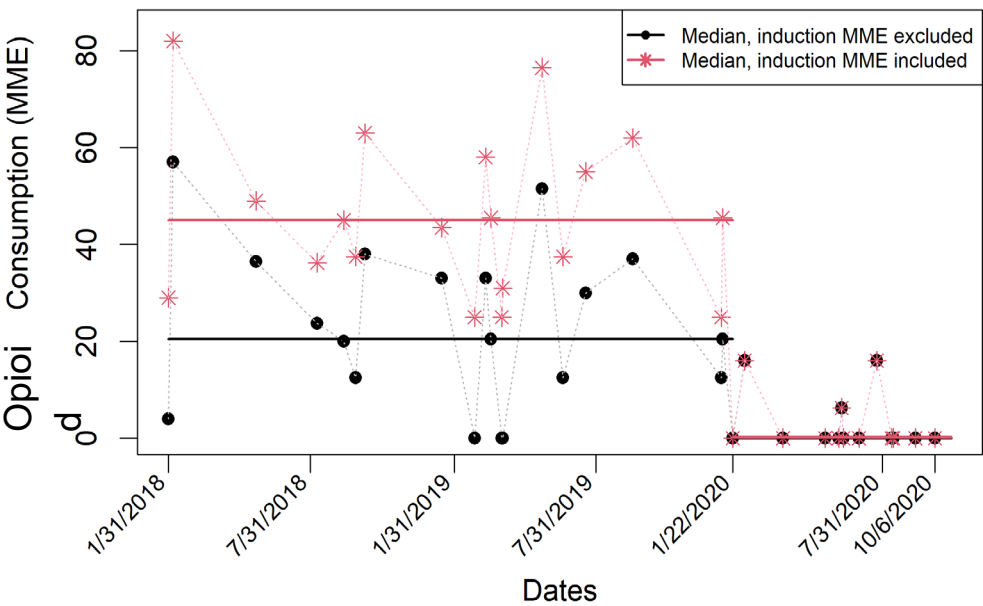

Supplement: Supplementary Figure 3 — Run chart for total oral morphine milligram equivalents (MME) in bone marrow donors (N = 32). The line represents the median MME. Induction opioids are included for all patients as noted by the red data points. Induction opioids are excluded as noted by the black data points. [file Image_3.PDF]
