# Supplementary material for: Decreased Opioid Consumption in Bone Marrow Harvest Patients Using Quadratus Lumborum Blocks in a Standardized Protocol
Source: Front Med (Lausanne). 2022 Apr 26;9:862309. doi: 10.3389/fmed.2022.862309 (PMC9086676; doi:10.3389/fmed.2022.862309)
Supplement: Supplementary Figure 2 — Standardized protocol for patients undergoing bone marrow harvest. [file Image_2.PDF]

Supplemental Figure 2.

Pre-op

Bone Marrow Donor Identified

- Patient information given to preop RN from bone marrow coordinator to chart review/ phone interview
- Pt scheduled at appropriate location
- Anesthesia MD Liaison to call and finalize preop & obtain consent
    - Discuss GETA vs Spinal & QL blocks
    - Clears until 5 AM (or 2 hours prior to OR time)
- RAPS team notified of need for QLs the night prior

Holding

QL Blocks BL

- 0.375-0.5 % Ropivacaine 20 mL each side
- **Avoid opioids if possible**
- Sedation (midazolam, dexmedetomidine) per RAPS team

Labs per Hematologist to include

CBC and Type and screen

Multimodal Meds

- Scopolamine patch
- Gabapentin 300 mg po
- Acetaminophen 1 g po

Intra-op

Spinal + sedation

- 10 mg 0.5% isobaric bupivacaine
- T6 level necessary
- Spinal done on stretcher (using epidural/ spinal chair) then patient flips themselves prone on to gel rolls on OR table and verbalizes comfort
- Propofol for IV sedation (Precedex as alternate) – start propofol at 50 mcg/kg/min
- ETCO2 nasal cannula

GETA

- **Avoid opioids if possible and safe**
- Ketamine for induction as an alternative (0.5 mg/kg)

Other intraoperative recommendations

- Ketorolac 30 mg IV
- Acetaminophen 1 g IV (if not given PO in holding)
- Ondansetron 4 mg IV
- Phenylephrine drip if hypotensive
- Fluids: crystalloid vs colloid (unlikely to need blood products intraop.- discuss with heme prior to giving) – fluid choice per in room provider
- If prolonged case or large volume of fluids expected to be given, recommend placing Foley at start of case or straight catheterize at end of case (spinal or general)

Candidacy for spinal is dependent on Anesthesia attending preference, patient preference/comfort/anxiety, contraindications to a spinal and possibility of long harvest that would exceed duration of spinal (uncommon, goal duration of all harvests < 2hr)

Post-op

- **No opioids** written on PACU orders
- If opioids are deemed necessary by the PACU RN- they must page the Attending for the case to discuss prior to administering
- If spinal is preformed discharge criteria includes:
    - Patient must ambulate without assistance after resolution of spinal
    - Patient should void in PACU prior to discharge
        - If unable to void and reliable patient, must void within 6 hrs of discharge from PACU, if unable they must report to the nearest ER for evaluation of urinary retention
- If GETA: standard discharge criteria
- Labs per hematologist- they will decide if PRBC transfusion is indicated
- Additional PONV treatment if necessary

Postprocedural follow up

- Patient will be given PO oxycodone prescription per the Bone Marrow Harvesting MD
- Recommend scheduled acetaminophen and ibuprofen to start immediately after procedure
- Patient will receive f/u call from BMH coordinator on POD #1 , 1 week post op and 1 month post-op
- Anesthesia Liaison will follow up with patient in 1-2 weeks to discuss PO pain med usage, duration of QL block, satisfaction with spinal/GETA/QLs via standardized questionnaire
